# Recovery from COVID-19 and acute respiratory distress syndrome: the potential role of an intensive care unit recovery clinic: a case report

**DOI:** 10.1186/s13256-020-02481-y

**Published:** 2020-09-10

**Authors:** Kirby P. Mayer, Jamie L. Sturgill, Anna G. Kalema, Melissa K. Soper, Sherif M. Seif, Evan P. Cassity, Jimmi Hatton Kolpek, Esther E. Dupont-Versteegden, Ashley A. Montgomery-Yates, Peter E. Morris

**Affiliations:** 1 Department of Physical Therapy, College of Health Sciences, University of Kentucky, 204D Wethington Building, 800 Rose Street, Lexington, KY 40526 USA; 2 Division of Pulmonary, Critical Care and Sleep Medicine, College of Medicine, University of Kentucky, Lexington, KY USA; 3 College of Pharmacy, University of Kentucky, Lexington, KY USA

**Keywords:** COVID-19, Acute respiratory distress syndrome, Post-ICU follow-up, Physical function, Emotional health

## Abstract

**Background:**

In this case report, we describe the trajectory of recovery of a young, healthy patient diagnosed with coronavirus disease 2019 who developed acute respiratory distress syndrome. The purpose of this case report is to highlight the potential role of intensive care unit recovery or follow-up clinics for patients surviving acute hospitalization for coronavirus disease 2019.

**Case presentation:**

Our patient was a 27-year-old Caucasian woman with a past medical history of asthma transferred from a community hospital to our medical intensive care unit for acute hypoxic respiratory failure due to bilateral pneumonia requiring mechanical ventilation (ratio of arterial oxygen partial pressure to fraction of inspired oxygen, 180). On day 2 of her intensive care unit admission, reverse transcription–polymerase chain reaction confirmed coronavirus disease 2019. Her clinical status gradually improved, and she was extubated on intensive care unit day 5. She had a negative test result for coronavirus disease 2019 twice with repeated reverse transcription–polymerase chain reaction before being discharged to home after 10 days in the intensive care unit. Two weeks after intensive care unit discharge, the patient returned to our outpatient intensive care unit recovery clinic. At follow-up, the patient endorsed significant fatigue and exhaustion with difficulty walking, minor issues with sleep disruption, and periods of memory loss. She scored 10/12 on the short performance physical battery, indicating good physical function. She did not have signs of anxiety, depression, or post-traumatic stress disorder through self-report questionnaires. Clinically, she was considered at low risk of developing post–intensive care syndrome, but she required follow-up services to assist in navigating the healthcare system, addressing remaining symptoms, and promoting return to her pre–coronavirus disease 2019 societal role.

**Conclusion:**

We present this case report to suggest that patients surviving coronavirus disease 2019 with subsequent development of acute respiratory distress syndrome will require more intense intensive care unit recovery follow-up. Patients with a higher degree of acute illness who also have pre-existing comorbidities and those of older age who survive mechanical ventilation for coronavirus disease 2019 will require substantial post–intensive care unit care to mitigate and treat post–intensive care syndrome, promote reintegration into the community, and improve quality of life.

## Introduction

The emergence of the novel coronavirus, severe acute respiratory syndrome coronavirus 2 (SARS-CoV-2), has had a significant impact on patients, families, healthcare systems, and communities. On March 11, 2020, the World Health Organization officially declared the SARS-CoV-2 virus outbreak a pandemic, officially known as coronavirus disease 2019 (COVID-19) [1]. Patients diagnosed with COVID-19 have a broad range of presentations, from asymptomatic carriers to those with severe critical illness with pneumonia, acute respiratory distress syndrome (ARDS), and multiorgan failure. In this case report, we describe the trajectory of recovery in a young, healthy patient diagnosed with COVID-19 who developed ARDS. We suggest the importance of intensive care unit (ICU) follow-up clinics to treat patients surviving mechanical ventilation or long-term ICU stay for Covid-19.

## Case presentation

### Pre–COVID-19 diagnosis

Our patient was a 27-year-old Caucasian woman with a past medical history of asthma. Her medical history was otherwise unremarkable. She was employed in customer service, was living with her husband, and denied a history of smoking or illicit drug use. She first noticed symptoms of dry cough, body aches, and low-grade fever (day 0). Four days later, she was diagnosed with bronchitis at a local community urgent treatment care center. She was prescribed azithromycin, a bronchodilator inhaler, and a steroid. She reported improvement of symptoms initially, but within 2 days, she had notable shortness of breath with minimal exertion with progressive dry cough, pain, and perfuse sweating. She reported significant shortness of breath when attempting to eat. Subsequently, she decided to seek treatment at her local community hospital. On day 7, she was admitted for room air oxygen saturation reported between 84% and 88% and profound dyspnea. The result of her respiratory viral panel was negative. She continued to have respiratory compromise with increasing oxygen requirements. On day 8, she was intubated for acute hypoxic respiratory failure due to bilateral pneumonia and was transferred to the medical intensive care unit (MICU) at our academic medical center.

#### COVID-19 diagnosis and ICU clinical course

**Fig. 1:**
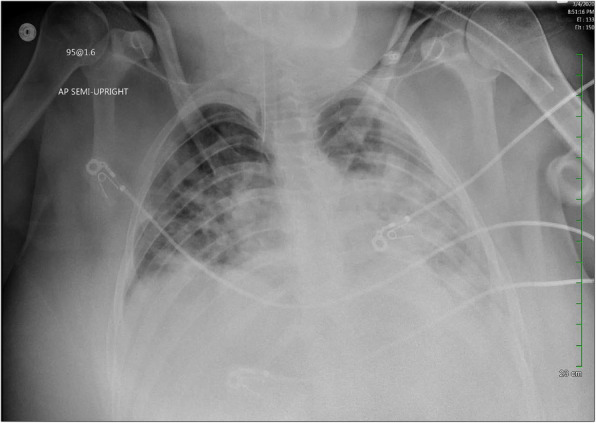
Chest radiograph obtained on day 1 of admission to intensive care unit revealed bibasilar airspace disease

**Fig. 2:**
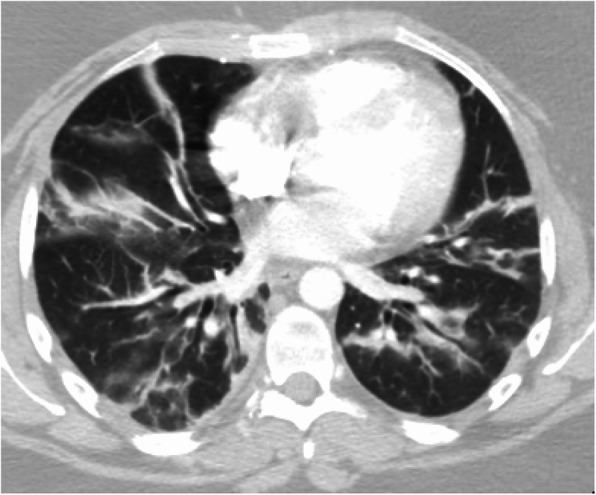
Chest computed tomography demonstrating bilateral lower lobe consolidation, volume loss with atelectatic changes in mild lower lung zones with scattered ground-glass opacities in upper lobe mostly central, consistent with bilateral pneumonia with lung volume loss and/or atelectasis

The patient remained intubated (70% fraction of inspired oxygen with positive end-expiratory pressure [PEEP] of 10 cmH_2_O and ratio of arterial oxygen partial pressure to fraction of inspired oxygen, 180) and minimally sedated (propofol and hydromorphone) for ARDS upon admission to the MICU (day 8). Her chest radiograph revealed bibasilar airspace disease (Fig. 1), and chest computed tomography demonstrated bilateral lower lobe consolidation and volume loss with atelectatic changes in mild lower lung zones with scattered ground-glass opacities in the upper lobe, mostly central, consistent with bilateral pneumonia with lung volume loss and/or atelectasis (Fig. 2). The patient’s Sequential Organ Failure Assessment score in the first 24 hours of ICU admission was 7. She was started on vancomycin, ceftriaxone, and azithromycin for empiric coverage of community-acquired pneumonia. Her brain natriuretic peptide and lactate levels were within normal limits, and aerobic and anaerobic blood cultures revealed no growth. The results of testing for urine legionella and streptococcal pneumonia antigens were also negative. The patient’s family reported no travel history or exposure risk, but, given the patient’s clinical symptoms, a test for COVID-19 was performed. A positive test result for COVID-19 was confirmed on day 9. In addition to ARDS, she met the criteria for a diagnosis of acute kidney injury (AKI; Kidney Disease Improving Global Outcomes stage 1) on the basis of decreased urine output and elevated creatinine level (increased from 0.77 mg/dl on first day in ICU to 1.45 mg/dl within 24 hours). Her liver enzyme levels were within normal limits (C-reactive protein, 24.3 mg/L).

**Fig. 3:**
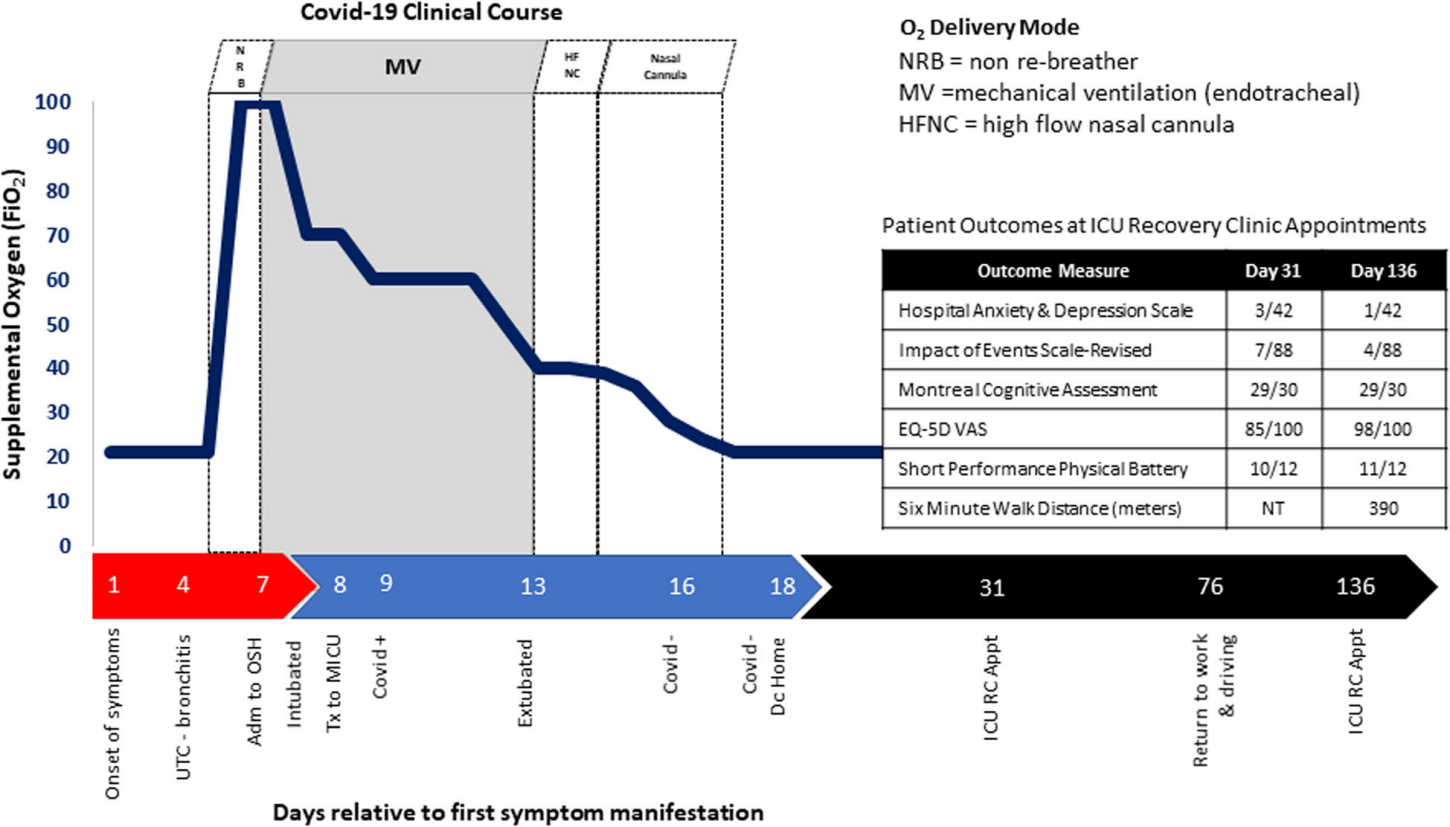
COVID-19 clinical trajectory demonstrating change in supplemental oxygen required from first onset of symptoms to follow-up in the intensive care unit recovery clinic with significant markers of patient case located on the horizontal axis. *Adm* Admission, *appt* Appointment, *Dc* Discharge, *FiO*_*2*_ Fraction of inspired oxygen, *ICU RC* Intensive care unit recovery clinic, *MIC* Medical intensive care unit, *NT* Not tested, *OSH* Outside hospital, *Tx* Transfer, *UTC* Urgent treatment center, *VAS* Visual analog scale

The patient’s clinical status gradually improved from days 9–12 of her illness. She tolerated weaning of oxygen and PEEP (Fig. 3). Her lung compliance was reported by her primary physician as “good” with low driving pressures (10–13 cmH_2_O). Her AKI improved with increasing urine output. On day 13, sedative medications were weaned fully, and the patient passed a spontaneous breathing trial. She was extubated to a high-flow nasal cannula. The patient’s respiratory status gradually improved with her being able to wean from supplemental oxygen on day 15. On day 14, physical and occupational therapy consults were performed, with the patient demonstrating modified independence with mobility and activities of daily of living. A physical therapist and an occupational therapist provided education on activity and exercise to promote strength and endurance. On day 16, the patient had a negative test result for COVID-19 by reverse transcription–polymerase chain reaction. A second test on day 18 confirmed the negative test result, and the patient was discharged to home from the MICU with her family later that afternoon.

### Postdiagnosis and ICU recovery clinic

On day 31, the patient returned to the ICU recovery clinic at our academic medical center. During her follow-up appointment, she complained of general fatigue and exhaustion. She stated she generally felt “wiped out.” She expressed minor difficulty with walking from the clinic garage, less than 500 feet from the clinic lobby due to fatigue. Physically, the patient participated in the short performance physical battery (SPPB), demonstrating a 0.82 m/second gait speed on 4-m habitual walk, 11.7 seconds to perform five times sit-to-stand testing, and > 10 seconds in tandem stance (total score 10/12), thus demonstrating a minor slowing in gait speed and minor difficulty with sit-to-stand performance, indicating minimal weakness in the lower extremities. The patient did have signs of mild depression (3/21 in the depression category of the Hospital Anxiety and Depression Scale [HADS]) related to her situation or memories of her illness, but her symptoms were not affecting her day-to-day life or preventing her from enjoying things she previously enjoyed. She did not have anxiety or signs of distress (HADS anxiety = 0/21, Impact of Events Scale–Revised 7/88). The patient did complain of short-term memory problems, which included no memory of her first 2 days in the ICU. She reported subsequent minor difficulty with daily short-term memory and word finding since her hospital discharge. She scored 29/30 on the Montreal Cognitive Assessment, missing 1 point removed for the cube copy test, which requires visual motor integration, depth perception, and spatial awareness. The patient also endorsed minor difficulty with sleep, including a few nights of disordered sleep with frequent waking. She reported a score of 85/100 on the EQ-5D visual analog scale.

On day 78, the patient reported returning to driving and return to work with modifications due to fatigue, including more frequent rest breaks, limited lifting of > 10 lb, and a stool to reduce time in prolonged standing.

On day 128 (approximately 4 months after discharge from the hospital), the patient returned to the ICU recovery clinic. She reported significant improvement overall, with only one episode of anxiety related to work that was alleviated with deep breathing strategies. The patient’s performance on physical, emotional, and cognitive outcome measures had improved (Fig. 3). The patient continued to endorse reduced endurance and periods of fatigue but was generally improved. At this time point, the patient performed the 6-minute-walk test (6MWD) with total distance ambulated 390 m, equating to 64% of her percent predicted 6MWD [2]. She also complained of insomnia, but she believed it to be unrelated to her ICU admission.

## Discussion

We present a case report of a young, previously healthy patient who developed ARDS due to COVID-19. The purpose of this report is to demonstrate the clinical trajectory and suggest the importance of ICU follow-up visits with objective physical battery testing as well as memory and cognitive testing. The patient responded well to supportive treatment in the ICU, including mechanical ventilation for 5 days and a total ICU stay of 9 days. On the basis of her Glasgow Coma Scale scores (ranging from 6 to 11), she never required deep sedation; in addition, on the basis of synchrony demonstrated with the ventilator and ratios of arterial oxygen partial pressure to fraction of inspired oxygen > 150, she did not receive neuromuscular blockers or undergo prone positioning for ventilation–perfusion matching. Upon awakening and extubation, she did have minor confusion with memory deficits, but she had no delirium noted by the primary physician. She did have AKI in the ICU, which recovered quickly. It is important to note that patients who develop AKI, especially when severe, with COVID-19 have increased secondary complications, including higher risk of death [3]. Our patient, however, continued to improve each day, with oxygen requirements gradually reducing, and she was discharged to home after two negative quantitative polymerase chain reaction test results.

Two weeks after discharge and 1 month after initial presentation of symptoms, the patient returned for her ICU recovery clinic appointment. Our ICU recovery clinic consists of a transdisciplinary team that includes a physician, an advanced practice registered nurse (APRN), a pharmacist, a physical therapist, and a social worker focused on improving quality of life after critical illness, organizing subspecialty follow-up care, and promoting rehabilitation. The primary objectives of ICU follow-up or recovery clinics are to address and treat post–intensive care syndrome (PICS) and help patients reintegrate into their societal roles following an admission to the ICU [4–7]. Post-ICU follow-up clinics also address traumatic emotional experiences of family members and engage all parties to optimize outcomes [8–11]. We are aligned with the national ICU recovery clinic movement organized by the Critical and Acute Illness Recovery Organization. Within this framework, we deploy a series of standardized evaluations for patients recovering from their ICU stay to assess emotional, cognitive, and physical health as well as health-related quality of life. Outcome measures assist in the development of the plan of care and referral for interventions such as post-ICU mental health interventions. Given our patient’s prior level of function (independent and employed), younger age, and scores on outcome measures, her trajectory of recovery is predicted to be strong. On the basis of her SPPB and quality of life data, she had already returned to nearly 80% of her baseline predicted physical function, except for slightly low gait speed, fatigue, and endurance during ambulation. The patient scored much higher (10/12 on SPPB) than previously reported data in an ICU follow-up clinic 1 month after ICU discharge (5/12 in 36 patients) [12]. She did not have signs or symptoms consistent with anxiety, depression, or post-traumatic stress disorder (PTSD). Compared with previously published ICU follow-up data [5, 12], the patient would be considered at low risk of developing PICS. However, considering her symptoms of reduced respiratory endurance and muscle weakness when performing the sit-to-stand test as well as her increased likelihood of anxiety related to isolation and frequent reminders of the pandemic [13, 14], she may benefit from continued ICU follow-up.

Our ICU recovery clinic physical therapist and our APRN educated the patient on exercise and activity at home to improve her functional status. Sleep, nutrition, and medications were addressed at both clinic appointments. The patient and her family were provided education on the importance of extensive hand hygiene and use of a face mask to reduce the likelihood of secondary transmission in the home and in public [15], especially given the reproductive number (R_0_) of COVID-19 [16]. In addition, education was provided on when to return to work and to driving, which focused on a gradual progression, such as practicing driving in areas of less traffic in and around her neighborhood and building up tolerance to full workload by starting part-time or modifying her physical requirements at work. The patient did report returning to driving as well as a modified work schedule about 2 months after hospital discharge. In addition, she was provided access to a shared ICU recovery email with all team members able to respond to inquiries, should she have questions about her recovery. The patient demonstrated significant improvements during her short-term recovery, but, even 4 months after discharge, she continues to have reduced endurance and exercise capacity (64% predicted 6MWD) and infrequent bouts of isolated anxiety and situational depression. Thus, the patient and the clinic team developed a short-term plan to have the patient continue to seek care in the ICU recovery clinic with a long-term goal to establish a primary care provider in her city of residence.

## Conclusion

We present this case report as a precursor to suggest that patients surviving COVID-19 with subsequent development of ARDS will require more intense ICU recovery follow-up. Even with her lower severity of illness and less complex ICU course, our patient will still have benefited from follow-up services and education to maximize her outcomes. We suggest that patients with a higher degree of acute illness who also have preexisting comorbidities and who are of older age who survive mechanical ventilation for COVID-19 will require substantial post-ICU care. Prior data of patients surviving acute respiratory failure suggest that ICU COVID-19 survivors will have substantial difficulties with anxiety, depression, PTSD, and physical disabilities as well as risk of secondary neurologic and cardiac complications [17–22]. Interdisciplinary follow-up care delivered using a recovery or PICS model will be of vital importance for positive outcomes in this population.

## Data Availability

N/A.
